# Learning to Use Artificial Teeth

**Published:** 1888-02

**Authors:** 


### Learning to Use Artificial Teeth.

Dr. Haskell, of Chicago, answers your New Brunswick man's question for information on the many causes which produce trouble in making plates fit, or stay in well. He describes the mouths well, but I think he should have gone on and described the *people* the mouths belong to, for instances: I would rather insert three sets for *three* women than one for *one man*. We ought to get three times the price for inserting a set for a man, that we do for a woman; then only under great pressure will I put in a set for a woman past seventy-five, and nothing would induce me to try it for a man. Again, take one who has been for years with only a few teeth; extract them, and insert a complete set, and if your patient is a man, he becomes at once a nuisance to his neighbors, and the horror of his poor, haunted dentist. I need not describe him further; the dentist who has been there will recognize the man at once. With such people I start with—"yes, I'll put you in a set, but you are going to have a terribly hard time before you get used to them."

I think most dentists make a mistake in leading their patients to believe that their plate is going to hold in so tightly *by suction* that they will be able to eat with it. I am confident no one ever ate a morsel, depending on the suction of a plate to hold it in place during the operation.

A patient asks me, "Shall I be able to eat with this set?"

"Yes—after you have learned how. You must remember that you have been without molars for a long time and the muscles of your cheeks and your tongue have forgotten the art of feeding the food to the teeth. Till they have re-learned the lesson of their youth, you cannot eat; furthermore, it is the muscles of the cheeks and lips that must learn to grip the teeth so firmly as to hold them in place while you are eating. This is very difficult at first, but finally becomes an involuntary act, and then your trouble ceases." No doubt, the worst thing for the dentist is for the patients to get it into their heads that the set is going to hold in so strongly by suction that they will be able to eat by that agency alone. The sooner the dentist succeeds in knocking that notion out of the patient's head the better for both.—*Cor. Items of Interest.*

